# Interpreting deep neural networks for the prediction of translation rates

**DOI:** 10.1186/s12864-024-10925-8

**Published:** 2024-11-09

**Authors:** Frederick Korbel, Ekaterina Eroshok, Uwe Ohler

**Affiliations:** 1 Max-Delbrück-Center for Molecular Medicine in the Helmholtz Association (MDC), Berlin Institute for Medical Systems Biology (BIMSB), Hannoversche Straße 28, Berlin, 10115 Germany; 2 Department of Biology, Humboldt-Universität zu Berlin, Unter den Linden 6, Berlin, 10099 Germany; 3 Department of Computer Science, Humboldt-Universität zu Berlin, Unter den Linden 6, Berlin, 10099 Germany

**Keywords:** Translation regulation, 5’ untranslated region, Massively parallel reporter assay, Deep neural networks, Explainable artificial intelligence

## Abstract

**Background:**

The 5’ untranslated region of mRNA strongly impacts the rate of translation initiation. A recent convolutional neural network (CNN) model accurately quantifies the relationship between massively parallel synthetic 5’ untranslated regions (5’UTRs) and translation levels. However, the underlying biological features, which drive model predictions, remain elusive. Uncovering sequence determinants predictive of translation output may allow us to develop a more detailed understanding of translation regulation at the 5’UTR.

**Results:**

Applying model interpretation, we extract representations of regulatory logic from CNNs trained on synthetic and human 5’UTR reporter data. We reveal a complex interplay of regulatory sequence elements, such as initiation context and upstream open reading frames (uORFs) to influence model predictions. We show that models trained on synthetic data alone do not sufficiently explain translation regulation via the 5’UTR due to differences in the frequency of regulatory motifs compared to natural 5’UTRs.

**Conclusions:**

Our study demonstrates the significance of model interpretation in understanding model behavior, properties of experimental data and ultimately mRNA translation. By combining synthetic and human 5’UTR reporter data, we develop a model (OptMRL) which better captures the characteristics of human translation regulation. This approach provides a general strategy for building more successful sequence-based models of gene regulation, as it combines global sampling of random sequences with the subspace of naturally occurring sequences. Ultimately, this will enhance our understanding of 5’UTR sequences in disease and our ability to engineer translation output.

**Supplementary Information:**

The online version contains supplementary material available at 10.1186/s12864-024-10925-8.

## Background

Regulation of mRNA translation enables rapid and local control of gene expression. As rate-limiting step, translation initiation is primarily controlled by the 5’ untranslated region (5’UTR) [1], which has a median length of 218 nucleotides in humans [2]. In it, regulatory sequence elements including RNA structural motifs and upstream open reading frames (uORFs) dictate the efficiency of translation. uORFs are present in 50 percent of human mRNA transcripts and conserved across species [3]. High-throughput genomics protocols that quantify translation have shown that uORFs are pervasively translated in a context-dependent fashion [4, 5], and that they both can promote or repress translation of the main ORF [6, 7].

Massively parallel reporter assays (MPRA) can be applied to measure the effect of vast numbers of designed and natural regulatory sequences on gene expression [8, 9]. A recent study built an MPRA library of entirely random 5’UTR fragments (i.e. uniform probabilities for A/C/G/U), assayed their effect on translation via the readout of mean ribosome load (MRL) observed in a human sample cell line, and used the resulting data to train a deep learning (DL) approach, convolutional neural network (CNN) [10]. While successfully capturing the relationship between the 50-nucleotide long synthetic reporter sequences and translation, the underlying biological features that the model utilized remain unknown.

Explainable artificial intelligence (XAI) is a rapidly expanding focus area that aims at understanding complex machine learning models, such as CNNs. Traditional methods (e.g. linear regression or decision trees) typically rely on human insights to extract adequate features from data. This requires domain knowledge, but also implies that feature importance can be read from the model coefficients directly. On the other hand, CNNs automatically extract patterns from complex data, simplifying the feature engineering step. However, DL models require post-hoc algorithms to explain model behavior since model weights may relate to abstract concepts not directly interpretable by humans. For instance, attribution methods uncover the input positions most relevant for prediction by computing position-wise importance scores, which explain prediction output with respect to individual input examples [11]. Complete explanation of model predictions is, however, complicated by assigning meaning to the input positions highlighted with attribution methods (cf. ‘the interpretation gap’, [12]). Regardless, recent developments that apply pattern recognition algorithms on top of explainability methods display a promising path towards closing the interpretation gap [13]. XAI therefore holds great potential for identifying novel biological mechanisms from functional genomics data.

**Fig. 1:**
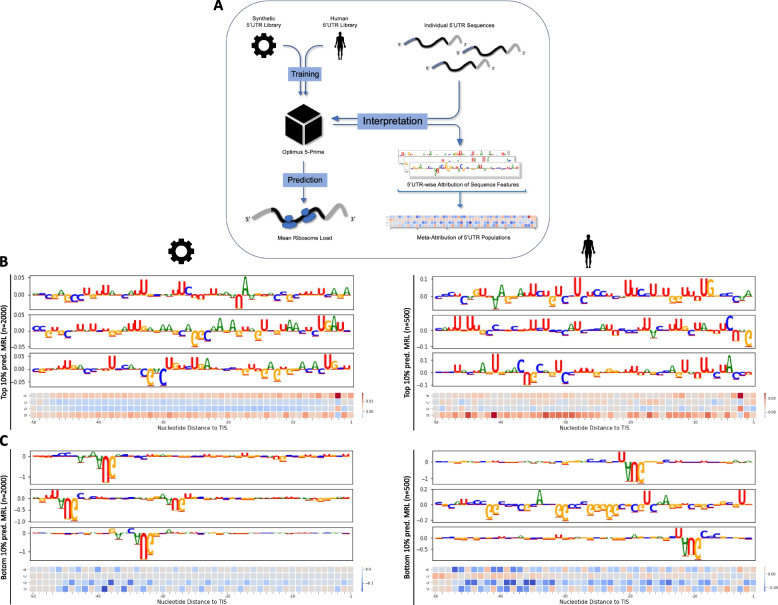
Extracting learned representations of regulatory logic. **a** A CNN predicts MRL of an mRNA given the sequence of its 5’UTR. We apply the integrated gradients algorithm on models trained on synthetic or human reporter data. From the attribution maps for individual 5’UTRs, we generate meta-attribution maps of 5’UTR populations. **b**, **c** Feature attribution of the synthetic model trained and tested on synthetic reporters (left) and a human model trained and tested on human reporter data (right). Meta-attribution maps of the 10 percent of 5’UTRs with highest (**b**) or lowest (**c**) predicted MRL are complemented by example attribution maps of three individual 5’UTRs from the respective population

To extract representations of translation regulatory logic, we apply integrated gradients [14], a feature attribution method, to both the original CNN fitted on the synthetic data (the “synthetic model”, dubbed Optimus-5-Prime by [10]) as well as a version that we trained on a second MPRA data set from the same study, consisting of human 5’UTR fragments (the “human model”, Fig. 1A). Moreover, we compare the underlying features driving translation prediction on synthetic and human 5’UTR sequences and make use of this knowledge to learn a model with superior performance.

## Results

### Model interpretation recovers known biological phenomena

We first computed meta-attribution maps by averaging feature importance values over a population of input samples to extract general features learned by the CNNs (Fig. 1A). Isolating features that contribute positively or negatively to the prediction outcome, this approach readily verifies current knowledge. Both models learn that the initiation context upstream of the main ORF start codon as well as around start codons within the 5’UTR affects mean ribosome load (Fig. 1B, C). Specifically, both CNNs learn that adenine or guanine bases at position -3 from the main ORF start codon increase mean ribosome load of the mRNA, which partly represents the Kozak consensus context observed in humans [15]. Moreover, high GC content in 5’UTRs is often associated with low levels of translation due to more stable secondary structures [2]. Both models capture this effect and positively attribute AU nucleotides while negatively attributing GC nucleotides (Fig. 1B) throughout the sequence.

**Fig. 2:**
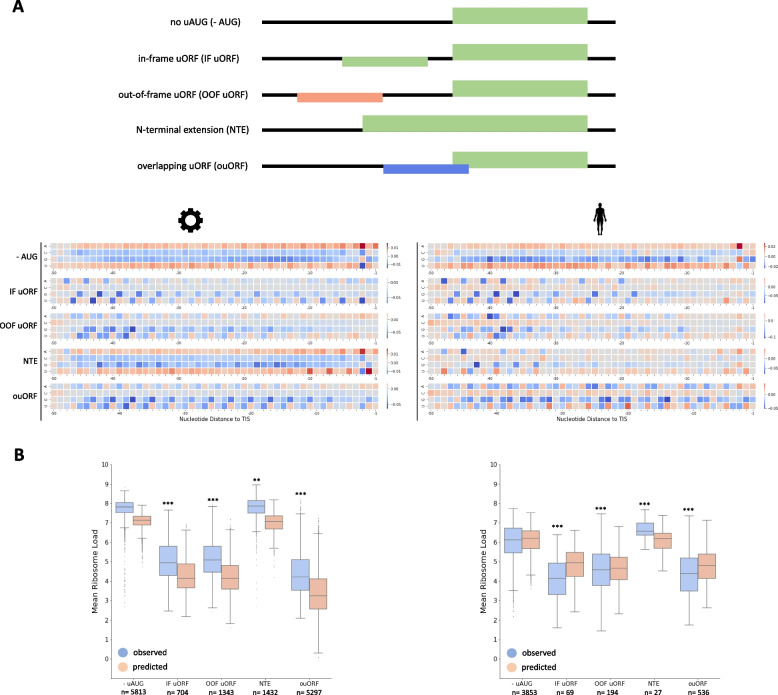
Meta-attribution of uORF-containing 5’UTR reporters reveals differential effects on translation. **a** Learned impact of putative translation in the 5’UTR on predicted MRL of the reporter mRNA for both models. Stratified by presence of one respective uORF or upstream start codon (uAUG) in the 5’UTR. **b** Observed vs. learned quantitative impact of upstream translation on MRL of the reporter mRNA for both models. Populations are the same as in A (unpaired two-tailed t-test; ** = *p* < 0.01; ***= *p *< 0.001)

Upstream ORFs regulate translation of the main ORF depending on position and nucleotide context of their start and stop codons as well as their reading frame [7]. Both human and synthetic-data models reproduce these previously studied regulatory features, such as a global repressive effect of upstream translation, while strongly attributing the nucleotide context of initiation codons (Fig. 1C). Furthermore, uORFs starting between -32 and -47 nucleotides upstream of the translation initiation site (TIS) were learned to be most repressive of ribosome load (Figs. 1C, 2A), as long as their stop codon was also located upstream of the CDS. Translation of these uORFs may lead to a lower efficiency of ribosomal re-initiation or decay of the mRNA induced by ribosome stalling [16, 17]. In addition, a regular periodic positive attribution of A/U nucleotides suggests that translation from near-cognate start codons is learned by the neural networks (Figs. 1B, 2A) [18].

To gain further insights into the effect of uORFs, we compared meta-attribution of reporters without upstream translation to reporters containing exactly one canonical upstream start codon (uAUG) or complete uORF (Fig. 2A). The meta-attribution of the synthetic model exhibits strongly negative effects, while interpretation of the human MRL model identifies a low number of uORFs/uAUGs in the context of naturally occurring sequences. Consequently, the synthetic model overestimates the negative effect of upstream translation (Fig. 2B). Nevertheless, both models distinguish between known regulatory mechanisms of upstream translation. For instance, in-frame uAUGs are not negatively attributed, since they can serve as an alternative TIS leading to an N-terminally extended protein product (NTE), which does not affect or may marginally increase ribosomal load of the coding sequence (Fig. 2A, B). On the other hand, translation at uORFs and overlapping uORFs (ouORFs) is observed to negatively impact ribosome load of the CDS (Fig. 2A, B), in line with current understanding [7].

### Synthetic data alone does not explain 5’UTR-mediated translation regulation in humans

Modeling mRNA translation with random synthetic 5’UTR sequences is attractive because of two reasons: First, it is unbiased, and in principle, the entire range of possible regulatory motifs is represented. Second, huge amounts of different random sequences can be generated. However, even an MPRA set of hundreds of thousands of sequences represents a tiny fraction of possible UTRs. When exploring the sequence space in an entirely random fashion, various characteristics of endogenous, functional, evolved genetic sequences may therefore be over- or underrepresented. In turn, this may lead to a model learning strong weights for features that rarely occur in biological systems, while not capturing other motifs under evolutionary selection. This is important to note, since variation in nucleotide composition of the 5’UTR has a profound effect on translation efficiency [19]. For instance, human 5’UTRs are depleted of uORFs located directly upstream the canonical translation initiation site and have a higher overall content of guanine and cytosine nucleotides associated with stable 5’UTR secondary structure (Figure S1) [3].

**Fig. 3:**
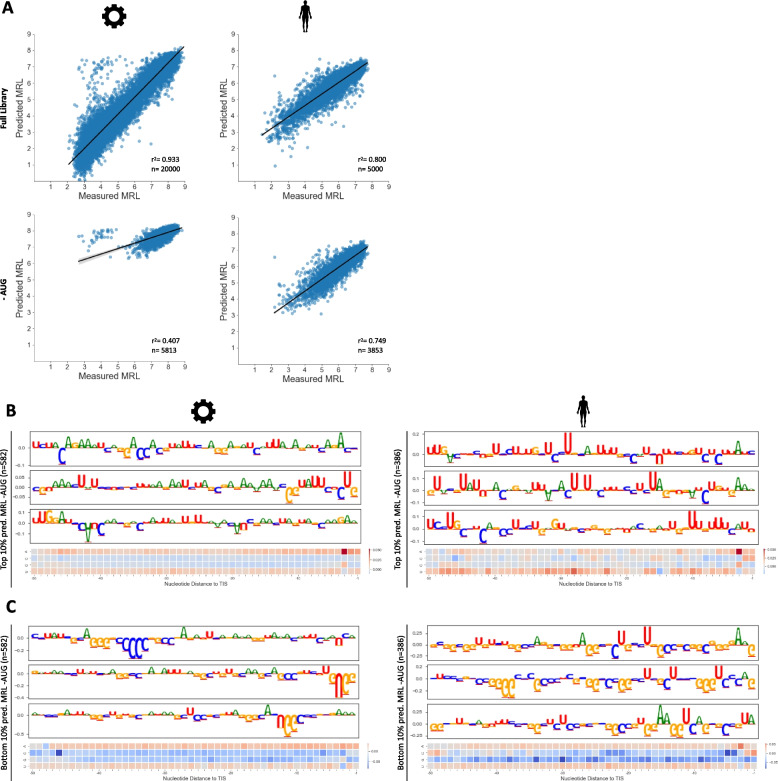
Occlusion of AUG-containing 5’UTR reporters reveals biases in randomly synthesized sequences. **a** Training and testing of the CNN on the synthetic (*n*=260,000) and human (*n*=20,000) reporter libraries and under exclusion of reporters with one or more AUG motif (-AUG). After removal of reporters with AUG in the 5’UTR, the performance of the synthetic model drops significantly, while it does not for the human model. **b**, **c** Meta-attribution maps for CNNs trained and tested on synthetic (left) and human (right) data under exclusion of reporters with AUG in the 5’UTR. A small number of reporters with a UG motif at the 5'end was excluded from the analysis (Figure S4). The 5’UTR reporters with highest 10 percent (**b**) and lowest 10 percent (**c**) predicted MRL are shown

We therefore aimed to reveal additional features by interpreting models trained after exclusion of reporters carrying one or more uAUG in their 5’UTR. This resulted in a significant drop in predictive performance for the synthetic, but not for the human MRL model (Fig. 3A). Additionally, almost no 5’UTR reporters with low to medium MRL were left in the synthetic data after removal of uAUG-reporters, suggesting that naturally occurring repressive motifs are underrepresented in synthetic 5’UTRs. This is supported by meta-attribution analysis, as a model trained on human 5’UTRs relies to a much greater extent on GC and U motifs, likely reflecting more stable secondary structure (Fig. 3B, C). Taken together, a model trained on the available synthetic data alone, i.e. on entirely random data that is sizeable yet covers a small amount of possible sequences, does not sufficiently explain 5’UTR-mediated translation regulation in humans.

### Fine-tuning on human 5’UTR data increases prediction performance

**Fig. 4:**
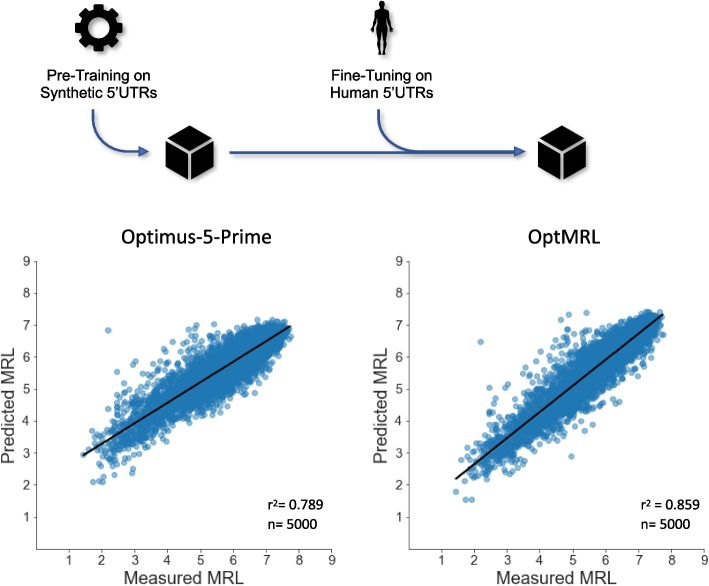
Developing the improved OptMRL model by training on synthetic and human 5’UTR data. The best-performing model was chosen after additional training of the synthetic model on human data (*n*=20,000) for 1-15 epochs. Performance of the synthetic model and an optimized MRL model (OptMRL) on a human 5’UTR reporter dataset (*n*=5,000, cf. Table S1). This approach reduced the prediction error on the human reporter library by one third

To generate a model that benefits from the larger sample size of synthetic 5’UTR reporter sequences yet being sensitive to features present in human 5’UTRs, we re-trained the synthetic model on human data (Fig. 4). For this, we filtered the 50-nucleotide human 5’UTR library by read counts similar to Sample et al. [10] to obtain 25000 samples, of which the 5000 reporters with highest read count across fractions were defined as test dataset and the remaining 20000 were used for training (Table S1). This transfer learning approach resulted in the OptMRL model, which more accurately reflects human 5’UTR sequence composition (Figure S2) while conserving performance on synthetic 5’UTRs (Figure S3). Importantly, it strongly improved on human data compared to the synthetic-only model, increasing prediction performance from 78.9 to 85.9 percent, thus reducing the error on the human 5’UTR reporter data by one third (Fig. 2E).

## Discussion

The ability to extract representations of learned features from “black-box” models of gene regulation enables us to dissect the behavior of models, to understand the characteristics of available data (cf. Figure S4), to put forth observations and hypotheses on gene-regulatory mechanisms, and to develop better models. Current developments in XAI aim to uncover interactions between individual features, and to shed light on higher order representations in the internal layers of the network. Yet, our work already demonstrates the impact of model interpretation for understanding and engineering RNA translation.

In this study, we show that random sequences suffer from an over-representation of certain features, such as uORFs and uAUGs and under-representation of other regulatory features, leading to poor model generalization to native human sequences. Two recent publications [20, 21] come to similar conclusions, albeit through different analyses.

We hypothesized that combining MPRA data from random and human sequences could enable models to better capture the translation phenotype of 5’UTR reporters. Iterative training on random and human MPRA data effectively covers a wider part of sequence space probed with random sequences, while fine-tuning weights on native human sequences occupying a smaller subset of sequence space allows the model to learn effects of more complex motifs unlikely to occur in random sequences. Consequently, OptMRL increases the predictive performance on a test set of human 5’UTR reporters compared to the original Optimus-5-Prime model while conserving performance on reporters with random 5’UTRs. While both models investigated in this study are limited to an input window of 50 nucleotides, we anticipate this approach to be a viable general strategy for building more comprehensive models of translation regulation from MPRA data. More advanced fine-tuning regimes than employed here may further improve model generalization to native sequences, but our results already provide an empirical argument for combining random and native sequences to better represent features of regulatory sequences.

A possible limitation is given by the generalization from MPRA-based translation readouts to endogenous measurements of translation. For instance, correlation between the MPRA-derived MRL and translation efficiency (TE) of native transcripts obtained from mRNAseq-normalized ribosome profiling counts in HEK293 cells is low (R = 0.0876, cf. Figure S5). Hence, CNNs trained on 5’UTR MPRA data poorly predict the translation efficiency of native transcripts [20, 21]. To a certain degree, this is expected, as 5’UTRs can reach a length of up to several kilobases compared to the maximum 100 nucleotides covered by the MPRA data. Native transcripts additionally vary in their CDS and 3’UTR. However, a lack of suitable data prohibits quantification of these individual contributing factors so far. Systematic investigation of MPRA readouts, MPRAs probing combinations of longer synthetic and native sequences as well as the relationship between 5’UTR and the remaining mRNA transcript will be central to deriving models that provide more meaningful predictions for native mRNAs.

Ultimately, this will promote our understanding of 5’UTR-mediated translation regulation, the role of 5’UTR mutations in disease as well as increase our ability to engineer desired translation phenotypes with synthetic regulatory sequences.

## Methods

### Data preprocessing

Datasets for computational analysis were downloaded from the Gene Expression Omnibus (GEO) under accession GSE114002 via the following link: https://www.ncbi.nlm.nih.gov/geo/query/acc.cgi?acc=GSE114002. Only reporters with a 5’UTR size of 50 nucleotides were considered in this study. Specifically, the unmodified eGFP library of synthetic 5’UTR reporters (GSM3130435) and the designed library containing human 5’UTR reporters (GSM3130443) were selected for analysis. Training and test datasets for the synthetic reporter library are the same as in Sample et al. [10] with a training set size of 260,000 and a test set size of 20,000. To assure direct comparability of the models, the preprocessing workflow of Sample et al. [10] was followed. Hence, the 5,000 human 5’UTR reporters with most sequencing reads across fractions were defined as test set and the remaining 20,000 human 5’UTR reporters were defined as training set.

### Model training and validation

All models investigated in this study follow the published Optimus-5-Prime architecture. They consist of three convolutional layers with 120 filters each, followed by a fully connected layer with 40 neurons and the output layer on top. If not stated otherwise, training hyperparameters were taken from Sample et al. [10]. Reporter-wise mean ribosome load values as target values for training of deep learning models have been adopted without change from GSE114002. As in Sample et al. [10], target values of training and test datasets were scaled separately using the scikit-learn [22] (v1.0.2) standardscaler before model fitting. Later, predicted target values were inverse-transformed accordingly. During training, loss on validation dataset was monitored after each epoch to review training history. The number of training epochs was determined manually based on the relationship between training and validation loss. All models were implemented in tensorflow [23] (v2.4.3), with indications for adopted code.

### Model interpretation

Model interpretation was performed with the integrated gradients algorithm [14], where changes in prediction outcome are measured for small changes in the input. In a custom python script, the integrated gradients were computed over a linear path from a null matrix as baseline to the actual input matrix in 50 steps. The obtained values were then scaled relative to the input matrix. Attribution maps were obtained through visualization of attribution matrices as sequence logos, with letter height representing the relative importance of individual nucleotide positions. Sequence logos were computed with code adapted from the concise package [24] and Ghanbari et al. [25].

To generate meta-attribution maps that summarize attributed features of large populations of input examples, attribution matrices from individual input sequences were added and normalized by sample size. Given a number (n) of feature attribution matrices (N), the respective meta-attribution matrix (M) is obtained through:

$$\begin{aligned} M=\frac{\sum _{i=1}^{n} N_{i}}{n} \end{aligned}$$

### 5’UTR reporter subgroups

To dissect the contribution of individual functional types of upstream translation on ribosome load, 5’UTR reporters were sorted into groups depending on their reading frame as well as the location of their downstream stop codon. We defined upstream open reading frames as a combination of an AUG codon and a downstream stop codon (UAA, UGA, UAG) within the same biological reading frame. A python script was used to sort and separate 5’UTR reporters into five groups. Reporters without uAUG in the 5’UTR were used as control group and those with more than one or ambiguous upstream translation signals were excluded from the analysis. Correspondingly, we distinguished between reporters with one non-overlapping uORF in-frame with the coding sequence (IF uORF), one non-overlapping uORF out-of-frame with the coding sequence (OOF uORF), one overlapping uORF (ouORF) as well as an N-terminal extension (NTE) of the protein product elicited by an in-frame uAUG.

### Iterative occlusion

In a two-step iterative process, subsets of 5’UTR reporters with certain features were removed from the training, testing and attribution workflow in order to reveal additional subtle sequence features. For both synthetic and human data, models were then fitted separately on the remaining data. In a first iteration, 5’UTR reporters with at least one uAUG were removed from the training and test datasets (-uAUG). After fitting a model on the remaining data, feature attribution was performed accordingly. As our XAI analysis led us to notice that the adapters used in [10] ended with an adenosine, reporters with ‘UG’ as the first two nucleotides in the 5’UTR were additionally removed from the datasets in the second iteration, (-uAUG -UG). Again, feature attribution was performed on the models fitted on the remaining data.

### Transfer learning

The optimized MRL model was obtained through a transfer learning approach. To this end, the Optimus-5-Prime model published by Sample et al. [10], trained on synthetic 5’UTR reporters, was re-trained on 20,000 human 5’UTR reporters. To determine the optimal number of training epochs, a search was performed over a space of 1 to 15 training epochs using keras-tuner [26] (v1.1.3). The best-performing model was then selected for further analysis (OptMRL, Table S1).

### Correlation analysis

Ribosome profiling counts normalized for library size (ORFspM) and mRNAseq counts (tpm) were downloaded from GEO (GSM1887643 and GSM1306496) [27]. A fasta file of corresponding 5’UTR sequences was compiled from the GENCODE genome GRCh38p14 annotation v44 with fiveUTRsByTranscript function from the GenomicFeatures R package [28]. The resulting 5’UTR sequences were matched with ORFspM and tpm counts via transcript ID and translation efficiency was defined as the ratio of normalized read counts (ORFspM/tpm) and subsequently log2-transformed. Only transcripts with ORFspM and tpm above 1 were considered for the analysis. Since there may be multiple ORFs on one mRNA, only the ORF with the highest detected ORFspM value per gene was kept. Pairwise alignment of 5’UTRs from reporters with 5’UTR from native transcripts with the biopython PairwiseAligner() [29] yielded 6100 sequence pairs with translation values in both libraries. Pearson correlation between MRL and log2TE of sequence pairs was calculated with the scikit-learn package [22].

### Statistics

Outliers are dotted. *P*-values are the result of two-tailed t-tests calculated with scipy [30] (v1.8.0). Sample sizes and *p*-values are indicated where relevant. Statistical annotation (Figure S1) was performed with the statannotations package [31] (v0.5.0). All boxplots display the median in their central part, interquartile range (25th/75th percentile) on the box edge, and 1,5x interquartile range as whiskers. The coefficient of determination, r-squared, was used as measure of performance throughout this study (e.g. how well are measured mean ribosome load values explained by the artificial neural networks).

### Code availability

The OptMRL model is available via kipoi: /www.kipoi.org/models/OptMRL. Code was developed in python (v3.9.10) and R. Code to reproduce analyses and figures is deposited on github and available under the following link: https://www.github.com/ohlerlab/mlcis. OptMRL model weights, jupyter notebooks and code can be directly downloaded there.

## Supplementary Information

Supplementary Material 1.

## Data Availability

Datasets and code including jupyter notebooks to train models and produce figures are deposited on github (https://www.github.com/ohlerlab/mlcis). OptMRL model weights can be directly downloaded from out github repository. The OptMRL model is additionally available via kipoi (https://kipoi.org/) for simple usage.

## Code availability

https://www.github.com/ohlerlab/mlcis.
