# Supplementary material for: Interpreting deep neural networks for the prediction of translation rates
Source: BMC Genomics. 2024 Nov 9;25:1061. doi: 10.1186/s12864-024-10925-8 (PMC11549864; doi:10.1186/s12864-024-10925-8)
Supplement: Supplementary file 1 — Supplementary Material 1. [file 12864_2024_10925_MOESM1_ESM.pdf]

Supplementary Information

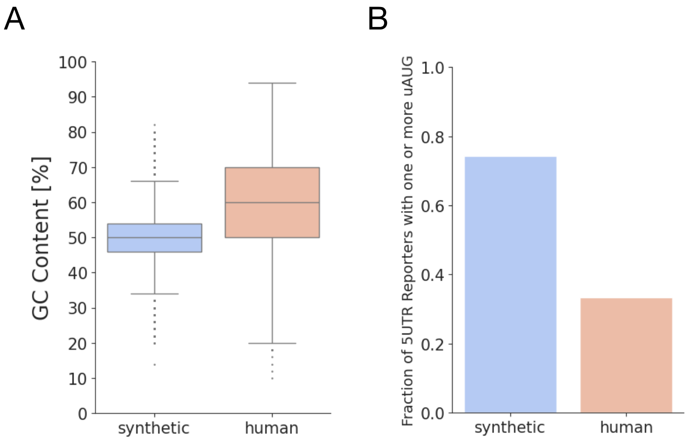

Fig. S1 Sequence Properties of synthetic and human MPRA libraries
Comparison between the synthetic 5'UTR library (n= 280,000) and the human 5'UTR library (n= 25,000) from Sample et al.. **(a)** GC content, which is indicative of secondary structure, is higher for human 5'UTRs than for random synthetic 5'UTRs. **(b)** The fraction of 5'UTRs with one or more uAUG is lower for the human 5'UTR dataset than for the synthetic 5'UTR reporters. Upstream AUGs are depleted in human 5'UTR sequences.

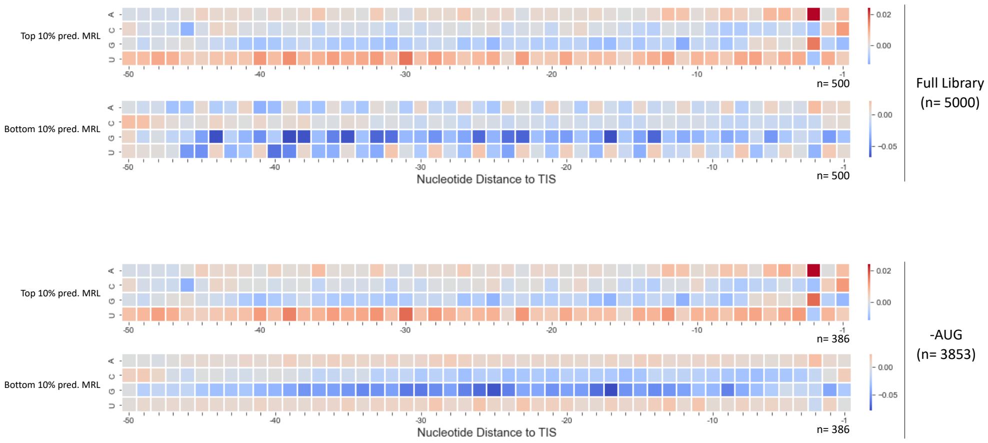

Fig. S2 Meta-attribution maps of the OptMRL model
Meta-attribution maps of the optimized MRL model for the full test library of human 5'UTRs and under exclusion of reporters with AUG in the 5'UTR. The model relies on general features of 5'UTRs when no AUG is present, predominantly on GC-rich patterns and U-rich patterns, i.e. likely related to secondary structure.

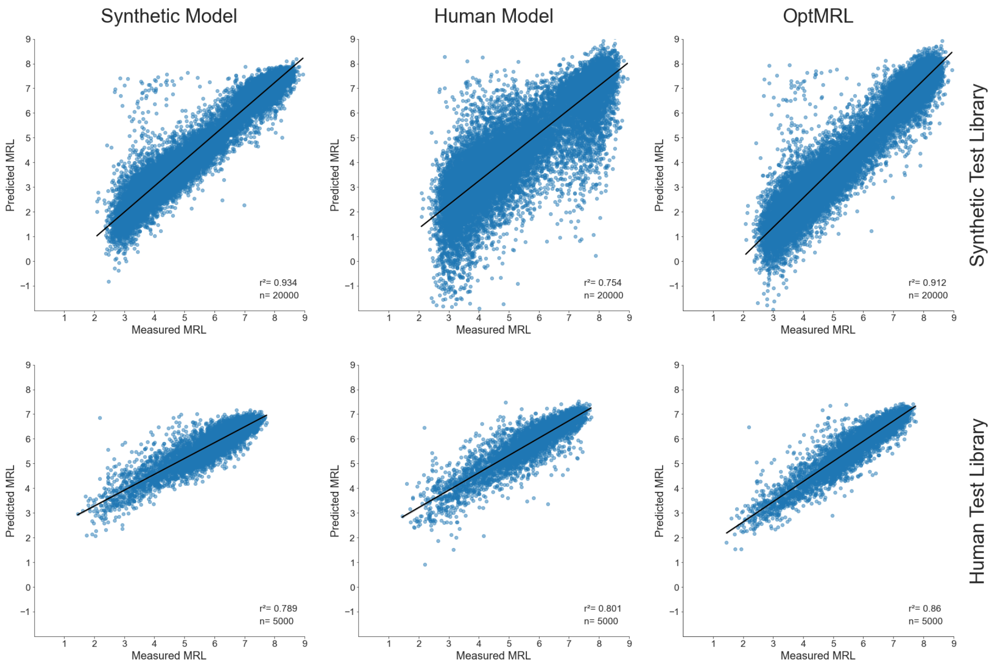

Fig. S3 Performance of three CNNs on synthetic and human MPRA libraries
The predictive performance of the synthetic model from Sample et al. [**?**], the human MRL model trained on 20,000 human 5'UTR reporters, and the optimized MRL model trained on 260,000 synthetic and re-trained on 20,000 human 5'UTR reporters, are compared on both a synthetic and a human test library. Although the human model performs better on a test library of human 5'UTR reporters than the synthetic model, performance is significantly lower on a synthetic 5'UTR test library. On the other hand, the optimized MRL model conserves performance on synthetic 5'UTR reporters, while increasing performance on human 5'UTR reporters compared to both other models.

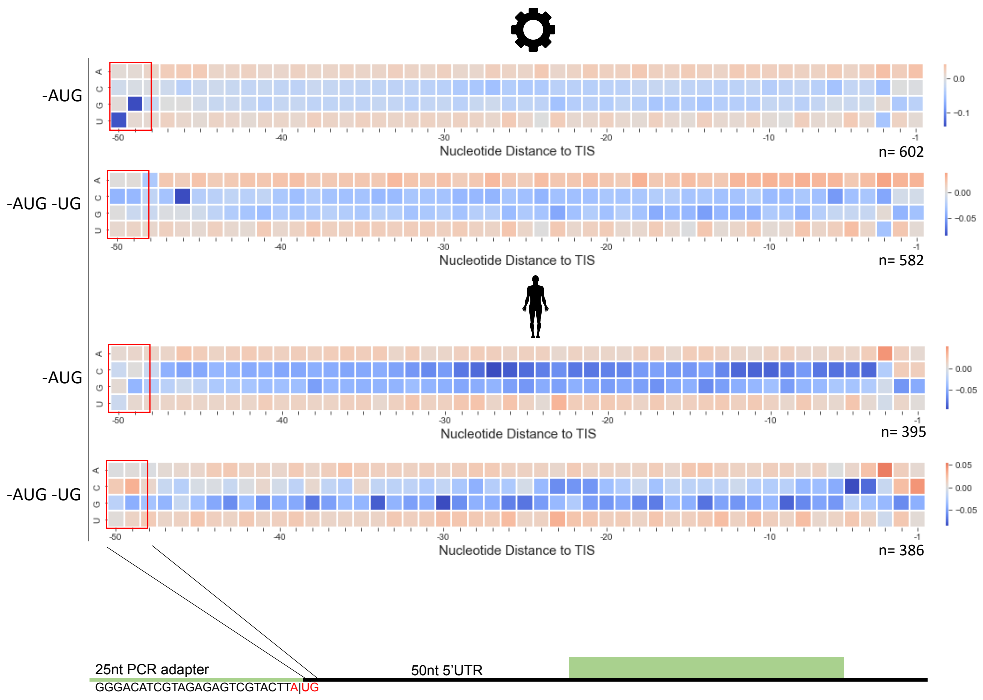

Fig. S4 Meta-Attribution reveals an experimental artifact
Meta-attribution maps of the 10 percent of reporters with lowest predicted ribosome load for versions of the model trained on the synthetic and human libraries, respectively. Models were trained and tested under exclusion of reporters with at least one upstream initiation codon (-AUG) and of those that exhibit 'UG' in the first two positions of their 5'UTR (-AUG -UG). A uridine and guanine as first two nucleotides in the 5'UTR of a reporter show strong attribution: together with the adenine in the last position of the PCR adapter, they create an AUG start codon in an alternative reading frame, resulting in a uORF overlapping into the coding sequence of those mRNAs. This artifact was learned by the model and is therefore revealed by feature attribution.

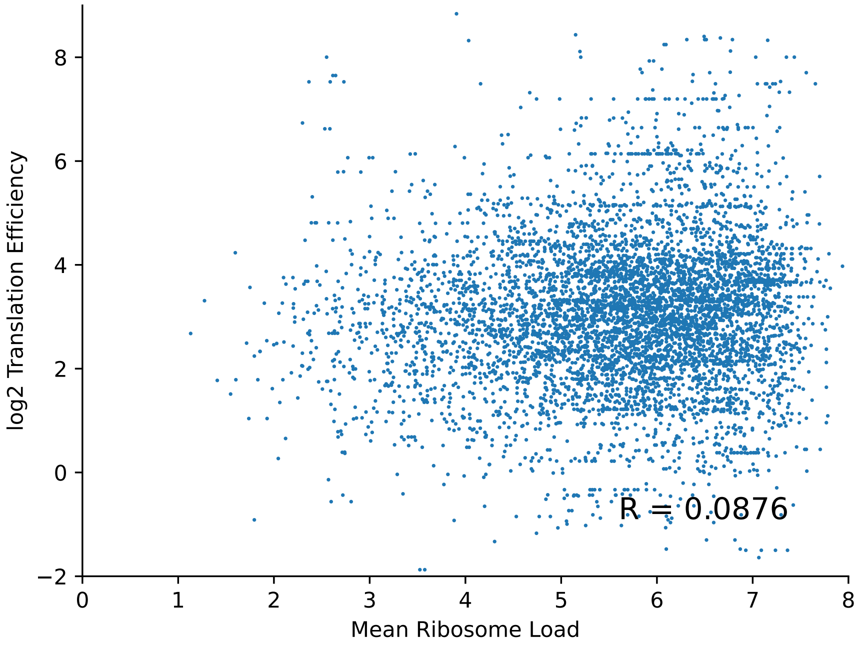

Fig. S5 Correlation of Mean Ribosome Load measured for 50nt human 5'UTR Reporters and Translation Efficiency of native whole-length Transcripts
Pearson correlation of MPRA-derived mean ribosome load and translation efficiency obtained from ribosome profiling and mRNA sequencing in HEK293T cells for 5'UTR sequence pairs. Pairwise alignment of 50 nucleotide long human 5'UTRs from Sample et. al MPRA dataset with ENSEMBL-annotated 5'UTRs of native transcripts from Calviello et. al yielded n=6100 sequence pairs.

Table S1 Overview of Model Training and Datasets

**Dataset**	**Training Size**	**Test Size**	**Model**	**Epochs**
synthetic (full)	260,000	20,000	O5P	3
synthetic (-uAUG)	122,927	6,014	MRL-uAUG	3
synthetic (-uAUG -UG)	118,525	5,813	MRL-uAUG-UG	3
human (full)	20,000	5,000	hMRL	9
human (-uAUG)	14,564	3,942	hMRL-uAUG	7
human (-uAUG -UG)	14,079	3,853	hMRL-uAUG-UG	7
synthetic + human (full)	260,000 (s) + 20,000 (h)	20,000 (s) + 5,000 (h)	OptMRL	3 + 13
